# Quantitative analysis of trafficking defects induced by heterozygous expression of hERG voltage sensor domain variants

**DOI:** 10.1080/19336950.2026.2672228

**Published:** 2026-05-15

**Authors:** Yihong Zhang, Joseph Carr, Jules C. Hancox, Stephen C. Harmer, Christopher E. Dempsey

**Affiliations:** a Translational Health Sciences, Bristol Medical School, University of Bristol, Bristol, UK; b School of Biochemistry, Biomedical Sciences Building, University of Bristol, Bristol, UK

**Keywords:** hERG, trafficking, heterozygous, dominant-negative, long QT syndrome

## Abstract

Long QT Syndrome Type 2 (LQT2) is a cardiac disorder caused by Loss of Function (LOF) mutations in the *KCNH2* gene that encodes the K^+^ channel hERG (K_v_11.1). Mistrafficking of hERG LOF variants is the dominant cause of LQT2. We recently characterized greatly attenuated cell surface trafficking in eight natural variants in a region of the hERG voltage sensor domain identified using evolutionary analysis. Here we have used quantitative On/In-Cell western assays to characterize trafficking of these variants under heterozygous conditions more relevant to the clinical circumstance. Dominant-negative effects of variant on wild-type (WT) cell surface expression and WT rescue of variant mistrafficking were separately assessed in co-expressions in which only WT or variant carried the HA-tag used to detect cell surface expression. Co-expression of all variants reduced cell surface expression and exhibited dominant-negative effects on trafficking. However, when compared to previous studies that utilized hERG glycosylation status as a measure of trafficking efficiency, the heterozygous effects on trafficking were smaller than expected. An identified relationship between pharmacological rescue of variant trafficking by the hERG blocker E-4031 and by co-expression with WT hERG indicated that inherent “rescuability” of variants might be characterized in advance of efforts to identify non-blocking trafficking rescuers.

## Introduction

The voltage sensitive potassium (K^+^) channel protein hERG (alternative nomenclature, K_V_11.1) is encoded by the *KCNH2* gene and carries the rapid delayed rectifier K^+^ current (I_Kr_) that makes a major contribution to the repolarization phase of cardiac action potentials (AP) [1,2]. Genetic variants that attenuate the function of hERG channels at the membrane surface of cardiomyocytes can result in inefficient AP repolarization, resulting in a lengthening of AP duration and the QT interval which underlies the LQT2 subtype of congenital Long QT Syndrome (cLQTS) [3–5]. For both LQT1 (loss of function of the voltage sensitive K^+^ channel KCNQ1 (K_v_7.1) [6]) and LQT2 [7], a dominant cause of loss of function is mistrafficking of newly synthesized protein (KCNQ1 or hERG), suppressing channel expression at the cell surface. A large proportion of LQT1/2 genetic variants are missense mutations of which more than 2000 are compiled in databases such as ClinVar [8] and gnomAD [9]. Only a small proportion has been classified as either benign or pathogenic in the ClinVar database [8] with the majority of LQT1/2 variants classified as Variants of Uncertain Significance (VUS) or otherwise unassigned.

**Figure 1.**
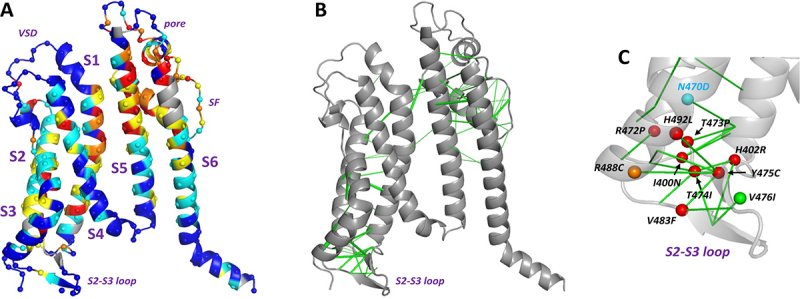
Trafficking and evolutionary coupling data mapped onto the membrane domain of a single subunit of hERG from Alphafold [18]. A, residue susceptibility to trafficking impairment in homozygous expression from a recent high throughput screen (HTS) [17] color-coded according to: red: trafficking highly intolerant to mutation; orange: low tolerance; yellow: conservative mutations accommodated; cyan: moderately exchangeable; blue: highly exchangeable. Grey ribbon: data not available. Data for seven residue positions around the S2-S3 loop are missing from the HTS set including for four variant positions studied here (see Table 1). B, equivalent view with high-probability evolutionary coupled residue pairs represented by green lines connecting Cα atoms. C, location of the set of natural variants in and near the hERG VSD S2-S3 loop that are characterized in this study. Amino acid Cα atoms are represented by red (severe mistrafficking phenotype); orange (mild mistrafficking phenotype) or green spheres (close to WT trafficking phenotype) as described in [16]. The location of variant N470D is also shown (cyan). Preparation of panel A is described in the Supplemental material (Suppl. Table S1 and Suppl. Figure S1).

Where characterized, mistrafficking of hERG [10] and KCNQ1 variants [6] results from perturbed folding of channel domains and/or assembly of channel subunit monomers into tetramers. This is expected to be followed by recognition of misfolded protein by the quality control systems of the endoplasmic reticulum (ERQC), protein retention in the ER and degradation [11,12]. We recently used Evolutionary Coupling (EC) analysis [13–15] to identify residue positions in cardiac voltage sensitive K^+^ ion channels likely to be susceptible to misfolding mutations based on the expectation that coevolution of amino acids identifies residue pairs whose interactions make significant contributions to the free energy of the folded state [16]. Loss of stabilizing contributions in nonconservative mutations involving EC residue pairs is proposed to shift the folding free energy toward poorly folded states to an extent sufficient to cause ER retention and degradation. A recent comprehensive High Throughput Screen (HTS) of membrane trafficking efficiency in hERG [17] is consistent with the expectation that non-conservative mutations at residue positions within the stable structured regions of ion channel proteins identified by EC analysis are likely to lead to misfolding and mistrafficking. Mutations in the conformationally mobile parts of the membrane domains associated with channel gating (especially the S4 helix and cytoplasmic halves of S5 and S6) are broadly resistant to misfolding/mistrafficking (Figure 1(A)). Trafficking competent mutants that lie within the mobile regions might, of course, be pathogenic due to perturbed gating or conductance.

Using EC analysis we identified a region in or near the cytoplasmic S2-S3 loop of the hERG Voltage Sensor Domain (VSD) likely to be susceptible to misfolding in non-conservative mutations (Figure 1(B)), and characterized eight natural variants with greatly attenuated trafficking [<20% of wild-type (WT) hERG] under homozygous expression using a quantitative *LI-COR*® based “On/In-Cell” assay [19] (Figure 1) [16]. In the present study we have extended the characterization of these variants to assess effects on trafficking under heterozygous expression (co-expression of WT and variant subunits), which mimics the autosomal dominant pattern of inheritance in LQT2 and better recapitulates the clinical phenotype. This is particularly important for hERG (and other multi-subunit cardiac K^+^ channels), as the coassembly of mutated subunits with WT subunits in heterotetramers may result in dominant-negative effects on channel trafficking that reduce cell surface expression to an extent greater than expected under conditions in which WT and mutant subunits don’t interact.

To date, only Oliveira-Mendes et al. have analyzed the effects of hERG variants on trafficking under heterozygous expression using an assay (pHluorin based) that *directly* quantifies channel expression at the cell surface [20]. They found that when expressed heterozygously T74R and R835P, but not I96T or P1026L, significantly reduced channel surface expression [20]. In contrast, most previous studies of heterozygous expression of hERG variants have used glycosylation status to assess effects on channel cell surface expression. This is based on evidence that the fully glycosylated hERG subunit population (WT and/or mutant), identified as a 155 kDa high molecular mass band on western blots, is equivalent to the population trafficked to the cell surface [21–23]. To further explore defective trafficking under heterozygous expression we used our previously described “On/In-Cell” western assay [16,19] to directly measure cell surface expression and quantitate the impact of the eight S2-S3 loop variants (and two previously studied variants, N470D and L615F) on the surface expression level of hERG. Separate quantitation of cell surface expression of WT *or* variant under heterozygous expression identifies both dominant-negative effects of variant on WT hERG surface expression as well as the ability of WT hERG to rescue variant mistrafficking. Our analyses identify smaller dominant-negative effects of mistrafficking hERG variants on channel cell surface expression than previously reported using hERG glycosylation status as a measure of trafficking efficiency. We also show, using the well-characterized hERG blocker E-4031, that the surface expression of some hERG variants may be inherently rescuable and identifiable in advance of discovery of non-blocking pharmacological chaperones.

## Results

### Co-expression of HA-tagged VSD variants with HA-tagged hERG

All expression experiments were performed using HEK293 cells which have been widely employed in assessing hERG trafficking including in the recent comprehensive HTS screen of hERG missense variant trafficking [17]. Co-expression of HA-tagged WT and HA-tagged VSD variants (0.25 μg plasmid for each of WT and variant) was assessed to determine total WT and variant channel protein expressed at the cell surface (“On-Cell”), and the channel protein in all cellular compartments including the cell surface (“In-Cell”). In addition to the eight poorly trafficked variants on the cytoplasmic side of the hERG VSD, HA-tagged N470D (Figure 1C) and L615F (in the hERG pore domain) were included as controls for variants which have previously been characterized in terms of trafficking, pharmacological rescue and dominant-negative effects on WT surface expression [21,23,24]. This experiment (equal amounts of WT and variant plasmid DNA) relates to the circumstance of a heterozygous patient carrying a VSD missense variant and is formally equivalent to previous co-expression studies of hERG variants with cell surface expression assessed using western blots in which *total* protein was identified without distinguishing between WT and variant [*e.g*. [7,21]].

**Figure 2.**
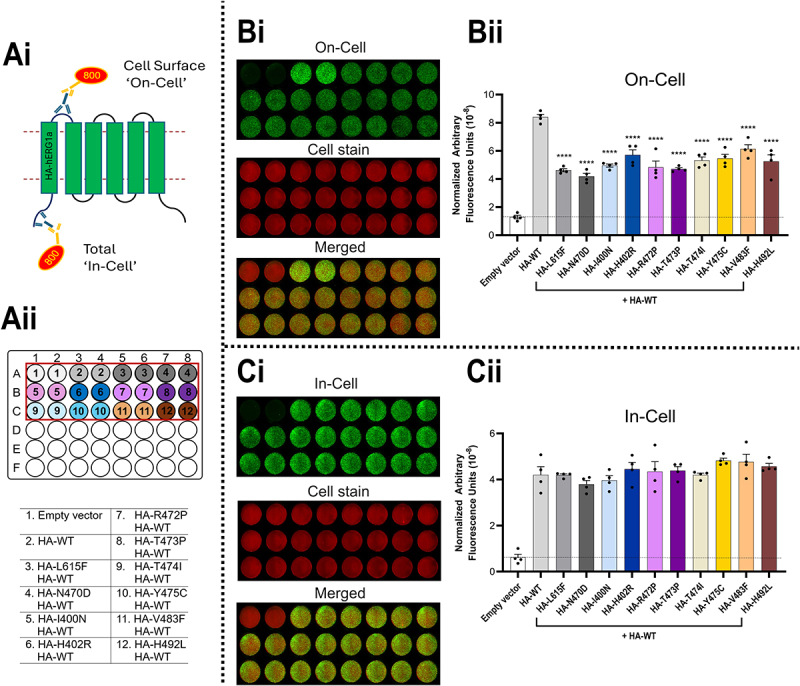
Heterozygous expression of trafficking-deficient variants with WT hERG reduces channel cell surface expression. The assay was performed 24 h after transient transfection. Ai, schematic of the experimental setup for *LI-COR*® based cell surface expression (“On-Cell”) and total cellular hERG expression (“In-Cell”) western assays. Aii, 48 well plate design and transfection conditions for the assays displayed in panels Bi and Ci. The total amount of plasmid used per transfection per well was 1 μg for all wells. Condition #1: 1 μg pcDNA3.1 empty vector; condition #2: HA tagged WT hERG1a (HA-WT) control: 0.5 μg HA-WT + 0.5 μg pcDNA3.1 empty vector; in all other conditions (#3–12): 0.25 μg HA-WT + 0.25 μg HA-tagged hERG1a variant + 0.5 μg pcDNA3.1 empty vector. B, “On-Cell” hERG cell surface expression. Bi shows a representative “On-Cell” western assay (green channel; top). To determine cell number, cells were stained using WGA-680 (red channel; middle panel) and the two channels are merged in the bottom panel. Bii, quantified “On-Cell” hERG cell surface expression levels of WT and variants co-expressed with WT. C, “In-Cell” hERG expression. Ci shows a representative “In-Cell” western assay (green channel, top), middle panel: cell stain. The two channels are merged in the bottom panel. Cii, quantified “In-Cell” hERG expression levels of WT and variants co-expressed with WT. Data are presented as (mean ± SEM) from four independent repeats (individual data points are displayed in Bii and Cii). Statistical analyses were performed using one way analysis of variance (ANOVA) and Bonferroni’s multiple comparison; **** = *p* < 0.0001 compared to HA-WT only. There are no significant differences in “In-Cell” channel expression levels (HA-variant + HA-WT vs HA-WT alone).

The results of co-expression of equal amounts of HA-tagged variant and HA-tagged WT hERG (HA-WT) plasmid are shown in Figure 2. Figure 2 also shows the strategy for separate identification of cell surface (“On-Cell”) and channel protein in all cellular compartments (“In-Cell”) (Figure 2Ai), and the distribution of individual assay measurements (Figure 2Aii) for identification of the sample wells in Figure 2Bi,Ci. These accompanying panels are omitted from subsequent “On-Cell/In-Cell” assay figures that assess the effects of heterozygous expression.

For all variants, co-expression resulted in considerable reduction of hERG channel protein (WT plus variant) at the cell surface (“On-Cell”) (Figure 2Bii). In each case the amount of channel protein measured “In-Cell” was broadly equivalent between HA-tagged WT (0.5 μg) and HA-tagged WT (0.25 μg) plus HA-tagged variant (0.25 μg), respectively (Figure 2Cii); this indicates that reduced cell-surface hERG expression (WT plus variant) is not a result of reduced synthesis or enhanced degradation of WT or variant when expressed under heterozygous conditions. Co-expression with WT of all variants that traffic poorly (each of the variants has < 20% of WT cell surface expression under homozygous conditions; see Figures 4A and 5A and Table 1), resulted in cell surface expression levels reduced to between 41% (N470D) and 68% (V483F) of WT alone values (Table 1). Although levels of WT and variant cell surface expression were not independently determined in this experiment, the relatively high residual cell surface expression indicates that any dominant negative effects are far from complete for any of the variants, including N470D and L615F. These data allow some comparison with previous data on co-expression of WT hERG with poorly trafficked variants. The previous data was obtained using western blot analysis based on the assumption that the 155 kDa high molecular mass, fully glycosylated hERG bands correspond to cell surface trafficked hERG whereas low molecular mass (135 kDa) partially glycosylated bands correspond to hERG retained within intracellular compartments. Western blot analysis of N470D/WT hERG co-expression has indicated near complete suppression of cell surface expression indicating a strong dominant-negative effect of N470D [23]. For hERG L615F, Anderson et al. reported an absence of fully glycosylated hERG on western blots in WT/L615F co-expression [21], which again differs from our observations of substantial (47% of WT) residual cell surface expression of WT/L615F (Figure 2).

**Table 1.** Cell surface expression of WT and hERG variants.

	Homozygous cell surface expression (HA-WT = 100)^a^	plus E-4031^b^	Total cell surface expression on coexpression (HA-WT = 100)^c^	Variant effect on WT surface expression (HA-WT + (un) WT = 100)^d^	Variant rescue by WT (HA-WT + (un) WT = 100)^e^	MAVE Homozygous cell surface expression (WT = 100)^f^
WT	100	186.8 ± 9.6				
L615F	6.4 ± 1.1	14.6 ± 1.3	46.9 ± 2.2	41.0 ± 4.2	35.3 ± 4.2	0
N470D	2.2 ± 0.6	38.2 ± 3.4	41.0 ± 3.6	33.9 ± 4.3	18.3 ± 2.8	NA
I400N	3.9 ± 0.5	61.5 ± 5.1	51.8 ± 1.9	38.8 ± 3.5	31.7 ± 4.4	0
H402R	12.9 ± 1.3	85.2 ± 5.2	62.8 ± 6.4	47.0 ± 6.4	43.3 ± 5.9	0
R472P	3.4 ± 1.0	15.2 ± 0.7	50.6 ± 7.5	47.9 ± 2.0	19.5 ± 2.8	NA
T473P	6.1 ± 1.4	6.7 ± 1.3	48.2 ± 2.0	34.1 ± 5.8	6.0 ± 1.4	0
T474I	8.3 ± 1.4	74.8 ± 3.3	57.2 ± 4.4	54.9 ± 5.0	39.9 ± 5.3	NA
Y475C	13.1 ± 1.9	43.0 ± 2.4	59.2 ± 4.9	46.6 ± 5.8	42.8 ± 6.0	NA
V483F	5.4 ± 1.4	46.8 ± 4.8	68.8 ± 5.7	63.1 ± 9.2	42.7 ± 4.2	0
H492L	17.5 ± 3.4	103.1 ± 11.3	56.5 ± 6.8	48.1 ± 3.9	51.9 ± 7.6	0

**Figure 3.**
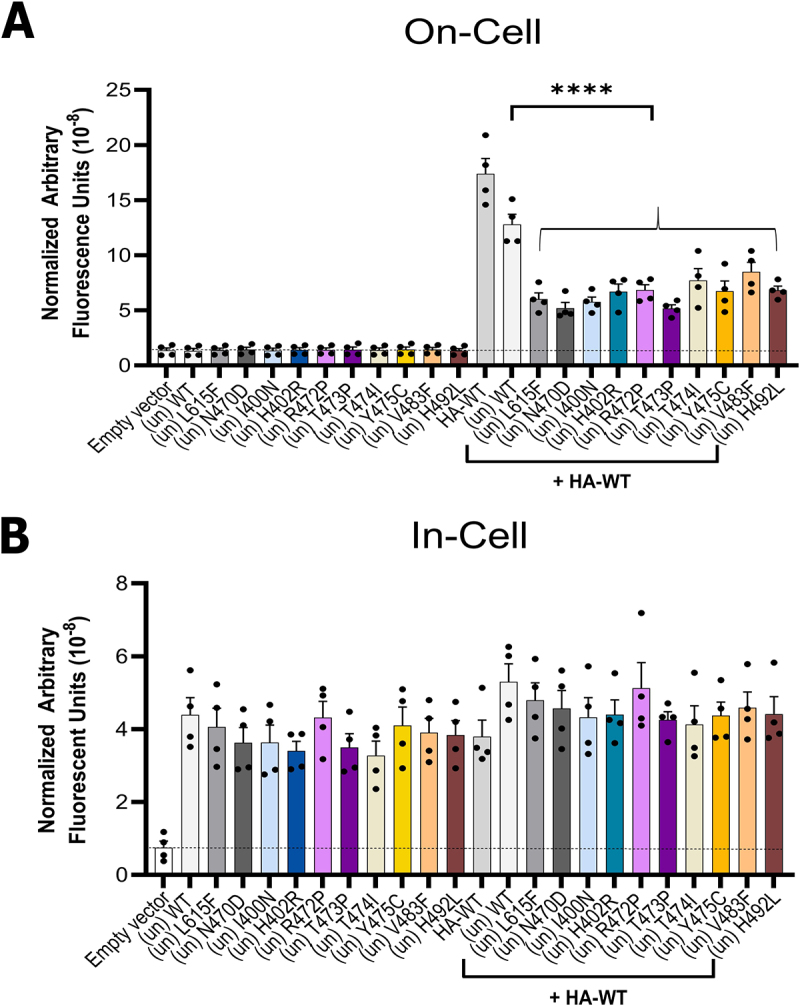
Untagged trafficking-deficient variants reduce cell surface expression of HA-tagged WT hERG. A, quantified “On-Cell” hERG cell surface expression levels of untagged (un) variants, HA-WT, and upon co-expression of HA-WT with untagged variants. B, quantified “In-Cell” hERG expression levels of untagged (un) variants, HA-WT, and upon co-expression of HA-WT with untagged variants. The assay was performed 24 h after transient transfection. The total amount of plasmid used per transfection per well was 1 μg for all wells. For empty vector (pcDNA3.1): 1 μg pcDNA3.1 empty vector; the untagged WT ((un) WT) alone condition: 0.5 μg (un) WT + 0.5 μg pcDNA3.1 empty vector; for untagged (un) variants expressed alone: 0.5 μg (un) variant + 0.5 μg pcDNA3.1 empty vector; for HA-WT alone condition: 0.5 μg HA-WT + 0.5 μg pcDNA3.1 empty vector; for untagged WT co-expressed with HA-WT: 0.5 µg (un) WT + 0.5 μg HA-WT; for the untagged variants co-expressed with HA-WT: 0.5 μg (un) variant + 0.5 μg HA-WT. Data are presented as (mean ± SEM) from four independent repeats. Statistical analyses were performed using one way analysis of variance (ANOVA) and Bonferroni’s multiple comparison; **** = *p* < 0.0001 compared to HA-WT + (un) WT. There were no significant differences in “In-Cell” hERG channel expression levels when compared to their respective controls: (comparisons made are either versus variant without HA-WT, or HA-WT alone).

**Figure 4.**
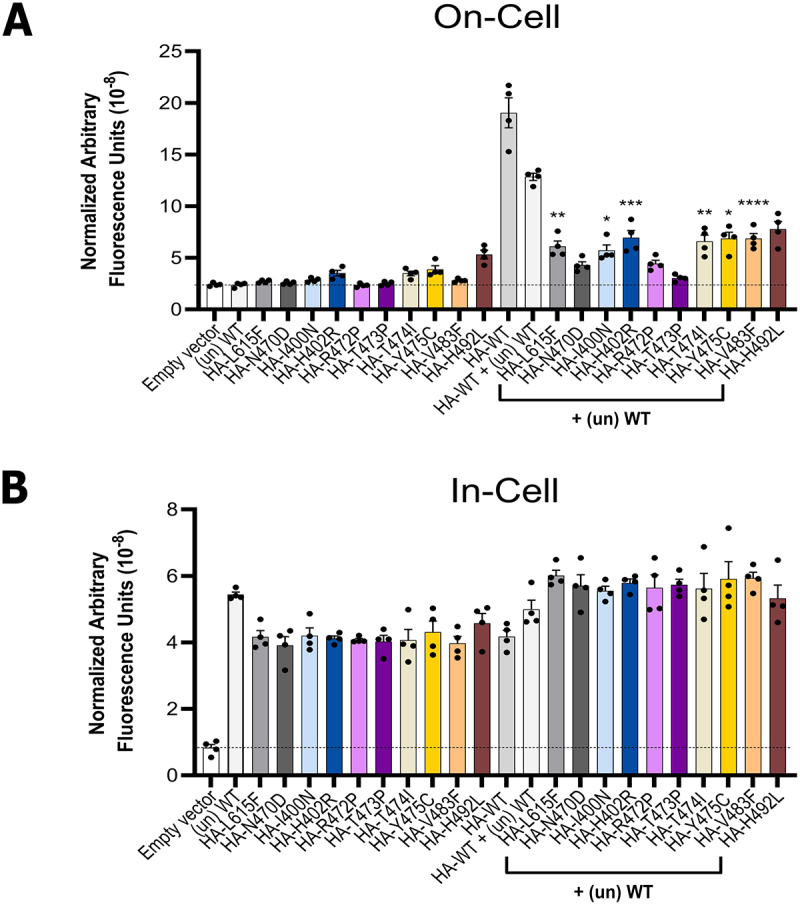
Co-expression of untagged WT hERG subunits promotes cell surface expression of trafficking-deficient variants to different extents. The assay was performed 24 h after transient transfection. A, quantified hERG “On-Cell” cell surface expression levels of HA-WT and HA-tagged trafficking deficient variants in the absence/presence of untagged WT hERG ((un) WT). B, quantified “In-Cell” hERG expression levels of WT and trafficking deficient variants in the absence/presence of untagged WT hERG ((un) WT). The total amount of plasmid used per transfection per well was 1 μg for all wells. For “empty vector”: 1 μg pcDNA3.1 empty vector; for (un) WT: 0.5 μg (un) WT + 0.5 μg pcDNA3.1 empty vector; for HA-tagged variants expressed alone: 0.5 μg HA-variant +0.5 μg pcDNA3.1 empty vector; for HA-WT alone: HA-WT 0.5 μg + 0.5 µg pcDNA3.1 empty vector; for HA-WT + (un) WT: 0.5 μg HA-WT + 0.5 μg (un) WT; for the HA-tagged variants co-expressed with untagged WT: 0.5 μg HA-variant +0.5 μg (un) WT. Data are presented as (mean ± SEM) from four independent repeats. Statistical analyses were performed using one way analysis of variance (ANOVA) and Bonferroni’s multiple comparison; * = *p* < 0.05, ** = *p* < 0.005, *** = *p* < 0.0005, **** = *p* < 0.0001 compared to variant in the absence of (un) WT (*e.g*. HA-L615F + (un) WT vs HA-L615F alone). No significant differences (*p* > 0.05) in “In-Cell” hERG channel expression levels were detected between HA-variants (with or without coexpression of (un) WT) when compared against HA-WT + (un) WT.

**Figure 5.**
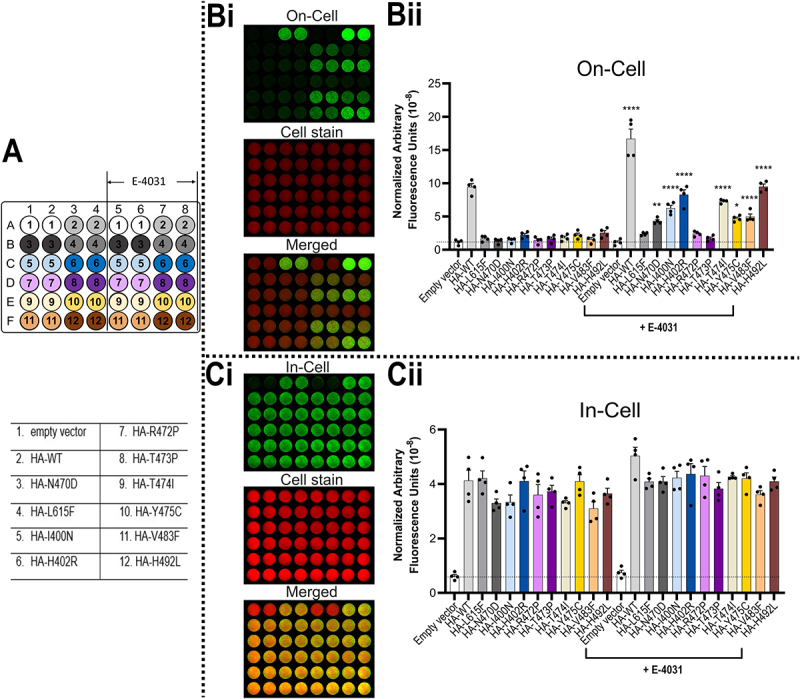
Rescue of variant trafficking by E-4031. A, diagram of the plate design and transfection conditions for the assays displayed in panels Bi and Ci. The total amount of plasmid used per transfection per well was 1 μg. For all HA-tagged WT or variants: 0.5 μg HA-WT or HA-variant + 0.5 μg pcDNA3.1 empty vector. Cells were transfected 48 h before assay and E-4031 (5 μM) was applied 24 h before assay. B, hERG “On-Cell” cell surface expression. Bi, representative “On-Cell” western assay (green channel; top). To determine cell number cells were stained using WGA-680 (red channel; middle panel) and the two channels are merged in the bottom panel. Bii, quantified hERG cell surface expression levels of HA-WT and HA-tagged trafficking deficient variants in the absence/presence of E-4031. C, “In-Cell” cellular hERG expression. Ci, representative “In-Cell” western assay (green channel, top), middle panel: cell stain; the two channels are merged in the bottom panel. Cii, quantified “In-Cell” hERG expression levels of HA-WT and HA-tagged trafficking deficient variants in the absence or presence of E-4031. Data are presented as (mean ± SEM) from four independent repeats (individual data points are displayed in Bii and Cii). Statistical analyses were performed using one way analysis of variance (ANOVA) and Bonferroni’s multiple comparison; * = *p* < 0.05, ** = *p* < 0.005, **** = *p* < 0.0001 compared to the corresponding value for HA-WT or HA-variant in the absence of E-4031. No significant differences (*p* > 0.05) in “In-Cell” channel expression levels were detected between HA-variants in the presence of E-4031 against HA-WT or HA-variant in the absence of E-4031.

Data is equivalent to cell surface expression levels (± SEM) presented in the relevant figures with the background (empty vector plasmid only) levels subtracted. ^a,b^Homozygous cell surface expression in absence (column 1) and presence of E-4031 (column 2) is from Figure 5. ^c^Total cell surface expression on co-expression of HA-WT plus HA-variant (Figure 2). ^d^Residual WT cell surface expression of HA-WT on co-expression with untagged (un) variant (Figure 3). ^e^Rescue of HA-tagged variant by co-expression with untagged (un) WT (Figure 4). ^f^Homozygous cell surface expression from MAVE data [17]; NA, no data in MAVE set. Significance (*p* values) can be found in the appropriate figures referred to in this legend.

### Co-expression of untagged VSD variants with HA-tagged WT hERG

An unambiguous assessment of the suppression of WT hERG trafficking by poorly trafficked variants (dominant-negative effects) can be obtained by co-expressing HA-tagged WT hERG with untagged variant. In this scenario, directly detected surface-trafficked protein corresponds exclusively to WT hERG since only WT hERG carries an HA tag. These data are shown in Figure 3A and Table 1 where levels of HA-WT protein reaching the cell surface are expressed relative to the HA-WT plus (un) WT control = 100%; (un) WT refers to untagged WT. Each of the poorly trafficking untagged VSD variants attenuated cell surface trafficking of HA-tagged WT hERG which was reduced to between 34% of control (by co-expression with untagged N470D or T473P), and 63% (co-expression with untagged V483F), once background non-specific fluorescence was accounted for (Figure 3A; Table 1). The degree of suppression of HA-WT cell surface expression by individual variants is broadly similar to the suppression of cell surface expression under conditions that both WT and variant are HA-tagged (compare Figures 3A and 2Bii). For example, the variants exerting the largest suppression of HA-WT cell surface expression in this experiment, T473P and N470D (Figure 3A), have large effects on channel surface expression in co-expression experiments in which both WT and variant are HA-tagged (Figure 2Bii). Similarly, V483F had both the smallest dominant-negative effect (Figure 3A) and the smallest effect on cell surface expression (Figure 2Bii). Levels of channel protein measured using the “In-Cell” assay are broadly equivalent when comparing WT or WT plus variant at equivalent amount of total plasmid (1 μg) (Figure 3B).

The data in Figure 3 reinforce the conclusion made in assessing channel cell surface expression in co-expression of HA-tagged hERG with HA-tagged variants (Figure 2), that while each of the variants reduced HA-WT cell surface expression substantially, none of the variants suppressed HA-WT surface expression completely. This differs from previous interpretations using western blot analysis of partially and fully glycosylated gel bands, in which for both N470D [23] and L615F [21] fully glycosylated hERG was negligible indicating near complete suppression of WT hERG surface expression.

### Rescue of cell surface expression of HA-tagged variant by untagged WT hERG

The reduction of HA-tagged WT cell surface expression by untagged variants (Figure 3) is consistent with the expectation that association of variant with WT protein can produce mixed WT-variant channel tetramers that traffic poorly. In contrast, observation of rescue of HA-variant trafficking by co-expression with untagged WT hERG would confirm that some mixed WT-variant channel tetramers can also reach the cell surface. These data are shown in Figure 4. We previously showed that trafficking of all of the VSD variants under homozygous conditions is inefficient [16], and this is confirmed here, including for N470D which wasn’t studied in our previous analysis (Figure 4A left hand series). Trafficking of many of the variants was partially rescued by co-expression with untagged WT hERG although rescue was significantly different from variant homozygous expression (*p* < 0.05) for only 6 variants (Figure 4A). However, this experiment demonstrates that for most of the variant co-expressions with WT hERG in which *both* WT and variant subunits are HA tagged (Figure 2) the cell surface channel subunit expression comprises a combination of WT and variant subunits. T473P, for which cell surface expression could not be detected under homozygous or heterozygous conditions is an exception, and for N470D and R472P any contribution of variant subunits to channel subunit cell surface expression is small.

### Pharmacological rescue of HA-tagged VSD variant cell surface expression with E-4031

Although enhanced trafficking of a selection of mistrafficked hERG variants by drug molecules thapsigargin [25] and lumacaftor (in human induced Pluripotent Stem Cell derived Cardiomyocytes; hiPSC-CMs) [26,27] has been reported, most successful pharmacological rescue of defective hERG variant trafficking has involved the use of hERG blockers [21,28–30]. hERG blocking molecules are unlikely to be developed into clinically useful drugs; however, they illustrate that hERG variant mistrafficking is potentially rescuable and provide a means of assessing trafficking rescue mechanisms. Since the hERG blocker E-4031 has been widely used to study pharmacological rescue of hERG variant trafficking [21,28], we measured E-4031 rescue of variant trafficking using the *LI-COR* based “On-Cell”/’In-Cell’ assays which affords a more quantitative assessment of rescue than has previously been achieved.

Most of the variants showed some rescue of trafficking (enhanced cell surface expression) in the presence of E-4031 (Figure 5). In contrast to previous studies using western blot analysis that reported the absence of effects of E-4031 on WT hERG trafficking [23,28,31,32], E-4031 produced a substantial increase in cell surface expression of WT hERG (by 1.8 fold) when measured using the “On-Cell” assay (Figure 5), which is similar to that observed in our earlier study of the hERG pore variant T634S [19].

**Figure 6.**
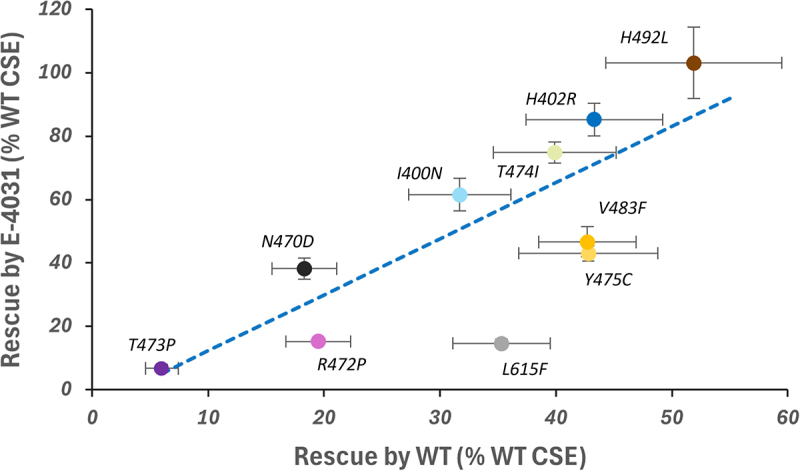
Rescue of variant cell surface expression upon co-expression with untagged WT-hERG or treatment with 5 μM E-4031. Rescue is represented as the percentage of WT-hERG cell surface expression (CSE) for each variant using the data in Table 1. Data points are colored according to the color scheme for variants used in previous data figures. The regression line was fitted for the VSD variants only (omitting L615F; see text) and has the following equation: y = 1.768x − 5.470; (R^2^ = 0.713).

When measured relative to the cell surface expression of WT hERG in the absence of E-4031, several VSD variants were rescued by E-4031 to levels close (H492L: 103% of WT) or similar (H402R: 85% of WT; T474I: 75% of WT) to WT hERG cell surface expression levels (Table 1). As previously reported [16] all hERG variants showed levels of channel expression in all cellular compartments as measured in the “In-Cell” assay that were similar to WT levels (Figure 5); there was no statistically significant effect of E-4031 on measured “In-Cell” levels of channel expression. For most of the variants there was a correspondence between the extent of rescue by WT-hERG and E-4031, so that variants that were more strongly rescued by co-expression with WT-hERG were also more strongly rescued by E-4031 (Figure 6). L615F was an outlier that was moderately rescued by WT-hERG but was poorly rescued by E-4031.

### Electrophysiological characterization of trafficked variants V476I and R488C

We previously identified two natural variants within the S2-S3 loop of the hERG VSD that showed moderate (R488C; 68.3% of WT) or high trafficking efficiency (V476I; 84.9% of WT) [16] (see Figure 1C). Efficient trafficking of V476I, a conservative variant, was unsurprising; however, R488C did not conform to the expectation in that study that a non-conservative mutation in a residue showing high probability evolutionary coupling should be destabilizing. Because of the high cell surface expression of these variants [16] we assessed their impact on channel function (Figure S2). While these analyses lie outside the main aims of this study, for completeness we provide them in the Supplemental material as a potential aid to variant classification. The most significant difference between these variants and WT hERG is in the reduced tail current density for R488C in a standard pulse voltage protocol (52% of WT) and in response to an action potential waveform (Figure S2). The observed reductions were similar to the reduced cell surface expression levels for R488C [16]. The small but non-significant reduction of peak tail current for V476I aligns with the small reduction in cell surface expression for this variant (84.9% of WT cell surface expression; [16]). Only small or non-significant differences in gating parameters were observed for both variants (Supplemental material, Table S2).

## Discussion

Our selection of natural hERG variants was based on the expectation that evolutionary coupling analysis might identify channel regions susceptible to misfolding with non-conservative amino acid mutations [16]. Characterization of a set of mistrafficking natural variants in and around the hERG VSD S2-S3 loop supports this expectation (Figure 1B,C) as does the comprehensive MAVE analysis of trafficking of hERG mutations (Figure 1A,B) [17]. Several of the VSD variants studied here are in short sequence regions where data is missing from the hERG MAVE set [17], but where equivalent data are available the mistrafficking S2-S3 VSD variants are also strongly mistrafficked in the MAVE set (Table 1). This indicates for these variants that the two independent methods of assessing homozygous variant trafficking efficiencies in HEK293 cells identify fundamental trafficking phenotypes.

Many hERG variants have been shown to exhibit dominant-negative effects on trafficking as assessed by glycosylation status [compiled in [21]] and/or function [22,23,33], and in some cases the reported dominant-negative effect is severe. By tagging only WT protein we demonstrate that each of the variants, including the previously studied N470D and L615F, attenuates cell surface expression of WT hERG indicating a dominant-negative effect (Figure 3). Some coassembly in mixed heterotetramers is confirmed by the finding that untagged WT hERG can partially rescue cell surface expression of most HA-tagged variants (Figure 4).

Quantitative interpretation of the trafficking data is complicated by the observation that co-expression of HA-tagged hERG with untagged hERG results in some suppression of cell surface expression of HA-tagged hERG (Figure 3). This observation implies some saturation of either the trafficking machinery or available sites at the cell surface, which limits detailed estimates of heterotetramer compositions in terms of numbers of variant subunits accommodated in a traffickable channel tetramer. It would be useful in future work to assess the nature of this saturation and determine whether saturation of surface expression is accompanied by saturation of macroscopic channel conductance over an equivalent range of WT plasmid concentrations. Despite this limitation, comparison of residual cell surface expression of both HA-WT hERG (Figure 3) and HA-variant (Figure 4) in co-expressions is consistent with a scenario for *most* variants in which heterotetramers containing one variant (3 WT/1 var) are trafficked to the cell surface, similar to an interpretation previously made for heterotetramers containing the mistrafficking variant A561V [22]. The inability of WT hERG to rescue trafficking of T473P indicates however, that heterotetramers containing even a single T473P subunit (3 WT/1 var) traffic poorly; there may also be some exclusion of T473P from channel tetramers. The same may apply to trafficking of heterotetramers involving WT co-expression with N470D and R472P since the apparent small degree of rescue by WT hERG for these variants (Figure 4) was not statistically significant. Overall, the experiments described in Figures 3 and 4 suggest that the channels detected at the cell surface when both WT and T473P (and perhaps WT and R472P or N470D) are tagged (as in Figure 2) are predominantly composed of WT subunits. For N470D this is consistent with a previous interpretation that the small current obtained in electrophysiological characterization of WT/N470D coexpressions arose largely from WT homotetramers, based on retention of WT-like activation parameters [23].

A notable difference between our data and some previous heterozygous expression studies concerns the relatively high levels of residual cell surface expression of hERG, whether measured under conditions where all subunits (WT and variant) are HA-tagged (Figure 2) or where only WT *or* variant was tagged under co-expression (Figures 3 and 4). Suppression of cell surface expression was far from complete for any variant, with cell surface expression in heterozygous expressions of around 34% of WT levels or greater when assessing only HA-WT cell surface expression (Figure 3, Table 1), or 41% or greater when total subunit cell surface expression (HA-WT plus HA-variant; Figure 2, Table 1) was measured. For some variants this may simply reflect a milder trafficking defect; T474I, for example, was previously shown to produce fully glycosylated bands on western blots when expressed in a stable HEK293 cell line with some functional expression measured electrophysiologically [3], consistent with the moderate cell surface expression observed here. However, an absence of fully glycosylated bands was previously reported for N470D [23] and L615F [21] when co-expressed with WT hERG, indicating essentially complete dominant-negative suppression of cell surface channel subunits; this differs from our observations for each of these variants. Some of this variation may arise from differences in experimental procedures. Huo et al. [31], for example, identified fully glycosylated subunits on western blots and some functional expression in co-expressions of G604S with WT hERG, whereas an absence of fully glycosylated subunits was reported in a different study for G604S-WT hERG co-expressions [21]. Some quantitative differences between cell surface expression for PAS domain variants measured by flow cytometry in comparison with cell surface expression assessed by glycosylation status has also been reported [34]. Significant current density in N470D-WT co-expressions in HEK293 cells at 37°C (around 30–35% of levels obtained in “rescue” experiments with E-4031) was previously observed while producing negligible levels of fully glycosylated bands on western blots [23]. Similarly, in a study of variant effects on functional expression of hERG [35] co-expression of hERG L615F with WT hERG produced around 25% of WT current density indicating at least this level of surface trafficking of hERG channels. These observations may suggest that in co-expressions involving poorly trafficked variants some channel subunits that are not fully glycosylated may reach the cell surface; unglycosylated hERG with glycosylation disrupted by an N598Q mutation has also been shown to traffic to the cell surface in *in vitro* expressions [36].

Our data suggest that it is unlikely that the reported absence of fully glycosylated hERG upon heterozygous expression of specific LQT2 associated variants (for example, N470D [23] and L615F [21]) results in a total loss of channel expression at the cell surface. Exactly what degree of loss of hERG channel function (whether due to defects in trafficking and/or gating) is required to cause QTc prolongation and increase the risk of Torsades de Pointes is an important question that warrants future investigation; this will likely be patient specific due to differing allele usage/expressivity and level of repolarization reserve. Importantly, even when not considering potential additional impact on channel gating, the observed mistrafficking-driven 50–60% reductions in cell surface expression under heterozygous expression (Figure 2) are significant and likely to contribute to the disease mechanism.

On/In-Cell assay-based assessment of hERG variant trafficking rescue by E-4031 provides a quantitative measure of “rescuability,” which differs from previous analyses in which variants have generally been classed either as rescued or not-rescued. An important observation is that trafficking rescue by E-4031 correlates with the rescue of variants by co-expression with WT protein, although E-4031 is a more effective rescuer (Figure 6). The similarities in trafficking rescue by E-4031 and by co-expression with WT hERG suggests that some poorly trafficking variants may be inherently rescuable, and that this might be established in advance of any future success in identifying non-blocking pharmacological trafficking chaperones.

Trafficking rescue by E-4031 arises directly from binding within the hERG pore cavity since rescue is attenuated by the same mutations of cavity amino acids that attenuate channel block [28]. Rescue is expected to result from the contribution of E-4031 binding energy to channel tetramer stabilization, with trafficking enhancement resulting from a shift in the unfolding-folding equilibrium toward the folded state. For 6 of the variants (N470D, I400N, H402R, T474I, V483F and H492L), the fold-enhancement of trafficking of between around 6 and 17-fold (Table 1) is equivalent to approximately -4.5 to -7.5 kJ.mol^−1^ per channel subunit, or an E-4031-hERG binding energy of -18 to -30 kJ.mol^−1^ per tetramer. These values are smaller than experimental measures of E-4031–hERG binding energies of around -42 to -46 kJ.mol^−1^ [37,38]. However, as described previously [28], folding and assembly of hERG subunits into channel tetramers that are stabilized by E-4031 occurs in the ER with a markedly different environment (*e.g*. high [K^+^] in the ER lumen and an absence of a [K^+^] gradient across the ER membrane) than the cell surface. A reduced binding affinity for E-4031 in these environments is the likely origin of the higher concentrations required for trafficking rescue in the ER compared to hERG block at the cell surface [28]. The hERG blocker binding site is also susceptible to mutations that allosterically affect the binding of hERG blockers [39,40]. L615F is poorly rescued by E-4031 (Figures 5 and 6; Table 1), and this may result from an attenuation of E-4031 binding by mutation in the conformationally-sensitive part of the channel pore where E-4031 binds.

Thermodynamic linkage of channel tetramer assembly with cell surface expression is also likely to be responsible for the enhanced cell surface expression of WT hERG by E-4031 observed here (Figure 5) and previously [19]. The identification of both partially and fully glycosylated hERG subunits on western blots under normal homozygous expression conditions *in vitro* indicates that a pool of non-processed WT subunits exists that can be pharmacologically chaperoned by shifting the folding/assembly equilibrium further toward assembled tetramers. This shifting of the folding/assembly equilibrium probably accounts for the enhanced cell surface expression of WT hERG by expression at reduced temperature (27°C rather than 37 °C) that is apparent when levels of cell surface expression are measured quantitatively [34]. Quantitation of E-4031 mediated trafficking rescue of variants in other parts of the hERG channel should allow a deeper understanding of thermodynamic coupling of hERG domains during channel folding and assembly.

## Experimental procedures

### Computational methods

Data mapping onto the hERG membrane domain structure in Figure 1 was done as follows. Evolutionary Coupling data were mapped onto a single subunit of an Alphafold structure [18] of the membrane domain of hERG using data from the Evolutionary Coupling server [41] as described previously [16]. Mapping of MAVE (Multiplexed Assays of Variant Effect) data from [17] utilized a MAVE data set encompassing the membrane domain in which synonymous and nonsense mutations were removed. Individual mutants were classified as either conservative or non-conservative [42,43] (see Suppl. Table S1) and were scored as either moderate to high trafficking (cell surface expression relative to WT hERG of 40% or greater) or poor (<40%) as described in [16]. A Python script was used to group mutant data for individual amino acid residues into the categories identified in the Figure 1 legend, based on the number of mutations at each residue position with trafficking either greater or less than 40% together with the number of mutations classed as conservative (Suppl. materials and Suppl. Figure S1).

Estimation of E-4031 binding free energies was made within a hERG folding (F)-unfolding (U) equilibrium model characterized by an equilibrium constant (K_FU_) in which folded subunits can traffic to the membrane surface as tetramers. Destabilizing mutations shift the equilibrium toward the unfolded state reducing trafficking competence; binding of E-4031 shifts the equilibrium back toward the folded state. E-4031 binding free energy can thus be estimated from the fold-increase in trafficking competence (equivalent to ΔK_FU_ in this model) resulting from E-4031 binding, and since E-4031 binds to channel tetramers, this binding free energy is distributed amongst four channel subunits. Within this model the binding free energy ΔG is (-RT.ln.ΔK_FU_) x 4 kJ.mol^−1^ at 37°C. An assumption is that the concentration of E-4031 (5 μM) is close to saturation and this has been established for E-4031 stabilization of hERG variant trafficking in HEK293 cells at 37°C [28]. Experimental E-4031:hERG binding energies were calculated from inhibitory constants (K_i_) from Diaz et al. [37] as described by Negami et al. [38].

## Experimental methods

### Mutagenesis

All HA-tagged hERG1a variants were generated as described previously [16] using QuikChange Site-Directed Mutagenesis (200519, Agilent Technologies, Stockport, UK). Full length untagged (un) WT hERG1a and variants were synthesized and inserted into pcDNA3.1(+) by GenScript Biotech Limited (Oxfordshire, UK). The HA-tagged WT hERG1a construct in pcDNA3.0 was a gift from Professor Alvin Shrier (McGill University, Canada) and contains the reference sequence of *KCNH2* (NM_000238.3, NCBI) with an extracellular HA tag inserted between the S1 and S2 domains [44]. The variant mutations were confirmed by sequencing the entire open reading frame (Eurofins MWG Operon, Ebersberg, Germany).

### Cell culture and transfection

HEK-293 cells (European Collection of Cell Cultures, Salisbury, UK) were maintained at 37°C, 5% CO_2_ in Dulbecco’s minimum essential medium with GlutaMAX-1 (DMEM; 31,966–021, Life Technologies, Paisley, UK). This was supplemented with 10% heat-inactivated fetal bovine serum (FBS, A5256801, Life Technologies, Paisley, UK) as previously described [16]. HEK-293 cells were not used beyond passage 30.

For “On/In-Cell” western assays, HEK-293 cells were plated on poly-L-lysine coated (P4707, Merck Life Science UK Limited, Dorset) Corning® CellBIND® 48-well Multiple Well Plates (CLS3338, Merck Life Science UK Limited, Dorset), and then transiently transfected using Lipofectamine 2000 (11668027, ThermoFisher Scientific, Paisley, UK) with a total of 1 μg of vector DNA per well. Each transfection condition was performed in duplicate.

### “On-Cell” (Cell surface expression) western assay

This assay was used to quantify HA-hERG channel expression at the cell surface (plasma membrane) as described previously [16,19]. Briefly, cells were directly incubated with primary antibody (mouse monoclonal anti-HA antibody (H9658, Merck Life Science UK Limited, Dorset)) diluted 1:1000 at 4°C for 1 hour. After washing, cells were fixed in 3.7% formaldehyde (252549, Merck Life Science UK Limited, Dorset) for 20 minutes, then stained with Wheat Germ Agglutinin 680 Alexa Fluor (W32465, Life technologies, Paisley, UK) at 5 μg/ml in HBSS for 10 minutes. After washing, the secondary antibody (anti-mouse IgG (H+L) (DyLight 800 conjugate) (5257S, Cell Signaling Technology, Leiden) diluted 1:1000 was added for 1 hour at room temperature. After washing, assay plates were scanned and analyzed using a *LI-COR*® Odyssey CLx imaging system.

### “In-Cell” (Total hERG protein) western assay

This assay was used to quantify the total amount of hERG channel detected in fixed and permeabilized cells as described previously [16,19]. Briefly, cells were fixed and then stained with WGA-680. After staining, cells were permeabilized using 0.1% Triton X-100 (Triton X100, Merck Life Science UK Limited, Dorset). The cells were then blocked for 30 min before adding the anti-hERG primary antibody (sc-377388, Insight Biotechnology Ltd, Wembley, UK) at a 1:1000 dilution for 1 hour. This antibody is raised against an N-terminal epitope (amino acids 96–270) which enables detection of both HA-tagged and untagged hERG. After washing, the secondary antibody (anti-mouse IgG (H+L) (DyLight 800 conjugate)) (5257S, Cell Signaling Technology, Leiden) was added at a 1:1000 dilution for 1 hour. After washing, assay plates were scanned and analyzed using a *LI-COR*® Odyssey CLx imaging system.

For the experiments presented in Figures 2–4, On/In-Cell assays were performed 24 hours after transfection. The On/In-Cell assays presented in Figure 5 were performed 48 hours after transfection, to enable pre-application of E-4031 for 24 hours.

### Analysis and normalization of “On/In-Cell” western assay data

A normalization by sum approach [45] was adopted. Raw arbitrary fluorescence intensities (700 and 800 channel values) obtained from the *LI-COR*® Odyssey CLx machine were exported into Excel (Microsoft). On a well-by-well basis the raw intensities for the 800 channel were normalized to that of 700 channel (WGA-680 cell stain) and the mean values for the duplicate technical replicates calculated. These were normalized to the total summed signal value of the 800 channel for that assay to obtain the normalized arbitrary fluorescence for each experimental condition. The mean ± standard error of the mean (SEM) was calculated from the four independent assay repeats as presented in the bar graphs in Figures 2–5. Individual data-points are superimposed on mean bar plots. Statistical analysis was performed on the normalized arbitrary fluorescence from all conditions in that set using ordinary one way ANOVA with Bonferroni post-hoc test for multiple comparison as appropriate. Details of the statistical test results are given alongside *p* values either in the main text or relevant figure legend. For full methodological details of the “On/In-Cell” Western assays and data normalization protocol please refer to [16, 19].

### Drug

E-4031 was purchased from Tocris (Abingdon, UK) and was made as a stock solution of 10 mM in distilled, deionized water. 5 μM E-4031 was applied 24 hours before assay as indicated in Figure 5.

## Supplementary Material

Supplemental Material

## Data Availability

All data supporting the findings of this study are available from the corresponding authors upon reasonable request.
