# Supplementary material for: Quantitative analysis of trafficking defects induced by heterozygous expression of hERG voltage sensor domain variants
Source: Channels (Austin). 2026 May 15;20(1):2672228. doi: 10.1080/19336950.2026.2672228 (PMC13182972; doi:10.1080/19336950.2026.2672228)
Supplement: Supplemental Material [file KCHL_A_2672228_SM5894.docx]

***Quantitative analysis of trafficking defects induced by heterozygous expression of hERG voltage sensor domain variants***

***Yihong Zhang^1^, Joseph Carr^1^, Jules C Hancox^1^, Stephen C Harmer^1^* Christopher E Dempsey^2^****

^1^Translational Health Sciences, Bristol Medical School, Biomedical Sciences Building, University Walk, Bristol, BS8 1TD.

^2^School of Biochemistry, Biomedical Sciences Building, University Walk, Bristol, BS8 1TD.

***Supplemental Material***

Document contains:

1. Description of mapping of hERG trafficking High Throughput Screen data onto the hERG membrane domain structure (Figure 1 of main paper).
2. Electrophysiological functional analysis of hERG voltage sensor domain S2-S3 trafficked variants V476I and R488C.

***Note:*** References cited in the Supplemental Material are compiled at the end of this document.

1. ***Mapping MAVE trafficking data onto hERG membrane domain structure***

***1.1*** ***Mapping incorporating mutant amino acid conservation (Figure 1 of main paper).***

A single subunit of the Alphafold structure for hERG (Uniprot identifier: Q12809) was downloaded from the Alphafold site ( https://alphafold.ebi.ac.uk/ ). The MAVE trafficking data from the Supplementary Information from [1] was edited to remove synonymous and nonsense mutants, and trafficking data for the membrane domain of hERG with short N- and C-terminal extensions (R394-R679, inclusive) was retained.

Each mutant in the MAVE set was assigned a value of 1 denoting a conservative mutation or a 0 (zero) for a non-conservative mutation. The sets of amino acids in the Table below were used to identify conservative mutations; any mutation involving amino acids within one of the sets was considered to be conservative. These sets were based on previous analyses of amino acid residue similarities [2,3] and particularly for the “hydrophobic” set, on the observations that Met and Cys are frequently found in mutatable pairs of hydrophobic amino acids within membrane domains, for example, in the hERG MAVE data; likewise, Ala and Thr are also frequently exchangeable within membrane domains.

***Table S1:* *Amino acid conservation sets for membrane domain***

| hydrophobic | aromatic | polar | negative | positive | low struct | A ↔T |
| --- | --- | --- | --- | --- | --- | --- |
| I | W | Q | E | K | S | A |
| L | Y | N | D | R | G | T |
| V | F | T |  | H | P |  |
| M |  | S |  |  |  |  |
| C |  |  |  |  |  |  |
| A |  |  |  |  |  |  |

A binary distinction between low trafficking (<40% of WT) and moderate to high trafficking (≥ 40% of WT) in the context of folding was used as described previously [4]. These criteria (conservative/non-conservative; low/high trafficking efficiency) were used to assign residue positions in the hERG membrane domain into the following groups using a Python script with priority in the order listed.

Highly exchangeable: 70% or more of residue mutations retain ≥ 40% WT trafficking. Blue in Figure 1 map.

Moderately exchangeable: 40-69% ≥ 40% WT trafficking. Cyan in Figure 1 map.

Zero mutants with trafficking ≥ 40% WT or one if this mutation is conservative. Red in Figure 1 map.

Only one (non-conservative) or two mutants with ≥ 40% WT trafficking. Orange in Figure 1 map.

Between 2 and 6 conservative mutants with ≥ 40% WT trafficking. Yellow in Figure 1 map

These criteria were chosen (i) to highlight the residues for which several conservative mutations within the hydrophobic set are allowed, (ii) to indicate that any residue for which 40% of mutants are able to traffic (equivalent to 7 or more of the 19 possible mutants at each residue) are at least moderately exchangeable, and (iii) to separate the residues with lowest exchangeability into “severe” (Red) and “poor” (Orange) categories. A small number of residues (11 out of 286) were not assigned to a group by these criteria and were assigned manually.

***1.2 Mapping using trafficking score averages.***

The analysis above was chosen to include amino acid conservation in the assignment of residue exchangeability. However, a mapping that similarly conforms to the expectation of low exchangeability of residues within the stable “scaffold” parts of the membrane domain identified by EC analysis [4] is obtained if the amino acids are simply grouped according to average trafficking scores without consideration of amino acid conservation. For example, Figure S1 compares the map of residue exchangeability incorporating amino acid conservation using the criteria described above, with a map based simply on the average trafficking score for all mutants at each residue position (“average residue trafficking score”) according to following criteria:

Average residue trafficking score (% of WT)

≥ 70 blue

40-69 cyan

25-39 yellow

10-24 orange

<10 red


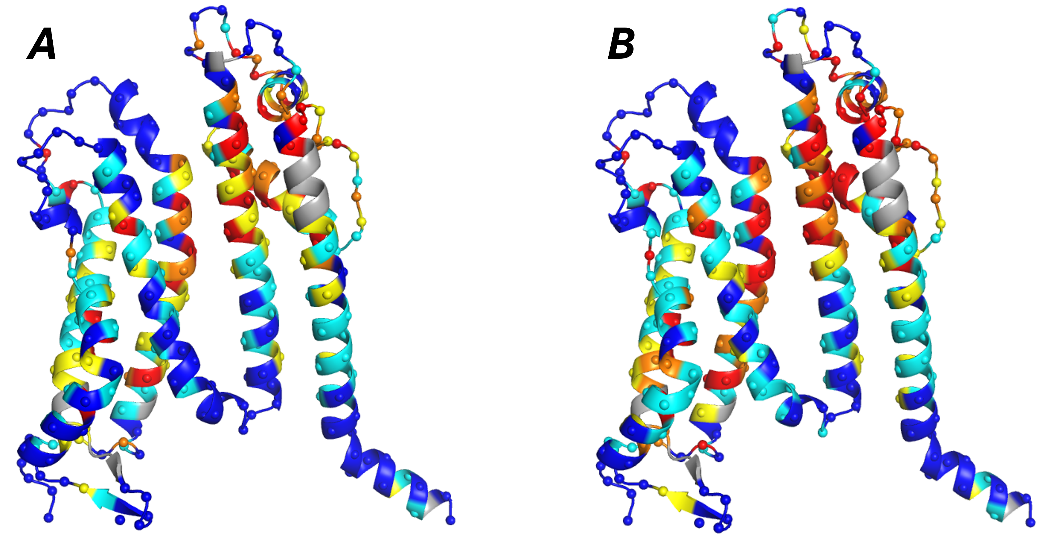


***Figure S1:*** Comparison of mapping of hERG MAVE trafficking data onto a single subunit of the Alphafold structure of the hERG membrane domain in which colour coding incorporates amino acid residue conservation (A) as described in section 1.1 above or was based on simple averaging of trafficking scores (B) as in section 1.2.

1. ***Electrophysiological characterisation of trafficked variants V476I and R488C***

***2.1 Methods***

For electrophysiology, HEK-293 cells were seeded into sterile plastic petri dishes and cultured until ~80% confluency was reached. Cells were then transfected using Lipofectamine 2000 with 1 µg hERG cDNA, and 0.3 µg CD8 cDNA in a pIRES vector (a gift from Dr I Baro, University of Nantes, France), as a positive transfection marker. This reaction mixture was left on cells for 16 hours to allow for sufficient transfection. Cells were then re-seeded onto 70% ethanol-treated sterile 13 mm glass coverslips and left for a further 24 hours to adhere. Successfully transfected cells were evident from relatively high binding of Dynabeads® to their membranes. All recordings were made 24-48 hours post-transfection.

***2.1.1 Electrophysiological recording***

I_hERG_ was recorded from transfected HEK-293 cells using the whole-cell patch clamp configuration, at physiological temperature (~37±0.5°C). Currents were obtained using an Axopatch 200B amplifier, alongside a CV-4/100 headstage and digitized with a Digidata 1320 interface system. Patch pipettes were pulled from non-filament borosilicate glass capillaries (outer diameter 1.65mm, inner diameter 1.20mm) to give a final resistance, when filled, of ~2-4 MΩ. The intracellular (pipette-filling) solution contained (in mM): 130 KCl, 1 MgCl_2_, 5 EGTA, 5 MgATP and 10 HEPES (pH was titrated to 7.25 with KOH). The extracellular (bath) solution contained (in mM): 140 NaCl, 4 KCl, 2 CaCl_2_, 1 MgCl_2_, 10 D-Glucose and 5 HEPES (pH was titrated to 7.45 with NaOH). To ensure temperature was controlled appropriately, extracellular solution was also directly perfused over the cells during recording by way of a home-built ‘switcher’ solution exchanger. The liquid junction potential between the bath and intracellular solution was deemed sufficiently small (+3.2 mV) and, as such, was not considered during analysis. Cell capacitance compensation was performed, and series resistance was routinely compensated between 65-80%. Data were acquired using a Bessel filter with bandwidth set at 2-10 kHz and an appropriate digitisation rate of 10-25 kHz used depending on the voltage protocol.

For action potential (AP) clamp recording, a simulated human epicardial ventricular AP was used as a command voltage, as described previously [5-8]. An interspaced P/4 subtraction was used to online leak correct the currents during this protocol. Leak subtraction was not used elsewhere.

***2.1.2 Data analysis***

To determine the slope factor (*k*) and half-voltage of activation (V_0.5_), the tail currents obtained during the standard current-voltage (I-V) protocol were normalised to the maximal tail current and plotted against test voltage. This relationship was then fitted with a Boltzmann function, as follows:

I = I_max_/(1+exp((V_0.5_-V_m_)/l))

Values for *k* and V_0.5_ of inactivation were determined from normalised I-V relationships obtained from the availability voltage protocol. These plots were then fit with the following function:

1-(1/1+exp[(V_0.5_-V_m_/*k*]))

Determination of the fast (τ_f_) and slow (τ_s_) components of hERG deactivation were calculated by fitting the tail current, elicited from the -40 mV step in the standard voltage protocol, with a biexponential equation:

I = A_f_ exp(-x/τ_f_) + A_s_ exp(-x/τ_s_) + C

Where I is the current amplitude at time point x. A_f_ and A_s_ represent the total current fitted by the τ_f_ and τ_s_ components of deactivation, respectively. C is the remaining current that could not be fit.

Time-course of recovery from the inactivated state was obtained from current plots over different durations, normalised to the maximal current. These relationships were then fitted with a one-phase decay function.

All data were normality tested to assess for normal distribution. Where this was passed, significance was assessed by way of a one-way ANOVA test. If normality testing failed, i.e. showed non-normal distribution, a Kruskal-Wallis test was used instead. Bonferroni correction was used *post-hoc*.

***2.2 Electrophysiology of trafficked variants V476I and R488C***

Figure S2 shows recorded current profiles from WT, V476I and R488C I_hERG_ in transiently transfected HEK293 cells; the currents were elicited by a standard protocol (shown as inset to panel A) composed of depolarizing voltage commands from holding voltage of −80 mV to +20 mV and then a repolarizing step to −40 mV before returning to holding voltage. WT I_hERG_ (left panel) exhibited well established characteristics: I_hERG_ developed during the applied depolarization, with a larger, resurgent ‘tail’ current elicited by the repolarizing step; the mean WT I_hERG_ tail density was 115.50±21.93 pA/pF (n=10). V476I and R488C I_hERG_ profiles were similar to WT, but R488C I_hERG_ density was significantly reduced to 60.28±12.11 pA/pF (n=15, p<0.05), while the modest reduction in V476I I_hERG_ 92.57±13.72 pA/pF (n=17) did not attain statistical significance (p>0.05 vs control), as shown in bar graph in Figure S2, panel Bi). Tail current deactivation was fitted with a bi-exponential function to derive fast and

slow (τ_f_ and τ_s_) time constant values. The mean τ_s_ value (describing the slow component of deactivation) was smaller for V476I than WT, with respective values of 1.53±0.05 s (n=17) and 1.88±0.06 s (n=15) for WT, (p<0.05). τ_f_ did not significantly differ between WT and mutant I_hERG_ (not shown), nor did the proportion of rapidly deactivating tail current for either V476I or R488C vs WT (p>0.05 for both, one-way ANOVA with Bonferroni correction; Figure S2, panel Bii).

I_hERG_ activation was evaluated using a standard I-V protocol comprised of 2 s depolarizing voltage steps from −80 mV to potentials between −40 and +60 mV. Normalized I–V relations (Panel C) for I_hERG_ tails were used to compare voltage dependent activation of the channels, with Boltzmann fitting used to derive V_0.5_ and *k* values. Derived V_0.5_ and *k* values for WT I_hERG_ were −15.6±2.35 mV and 7.64±0.58 mV (n=12), for V476I these were -21.19±1.85 mV and 8.95±0.43 mV (n=13, p>0.05 for both V_0.5_ and *k* versus WT), while for R488C these were −12.99±1.77 mV and 8.36±0.71 mV (n=10, p>0.05 for both V_0.5_ and *k* versus WT). With an action potential command, the mean peak I_hERG_ density for WT was 97.89±20.44 (n=11) and for R488C I_hERG_ it was 53.80±11.42 (n=10, p<0.05 versus WT), without affecting the peak current voltage (see Figure S2 panel E and Table S2).

Voltage dependence of I_hERG_ inactivation (plotted as availability) and recovery from inactivation for both V476I and R488C were similar to WT (see Figure S2 Panel Di and Dii, and Table S2).

**
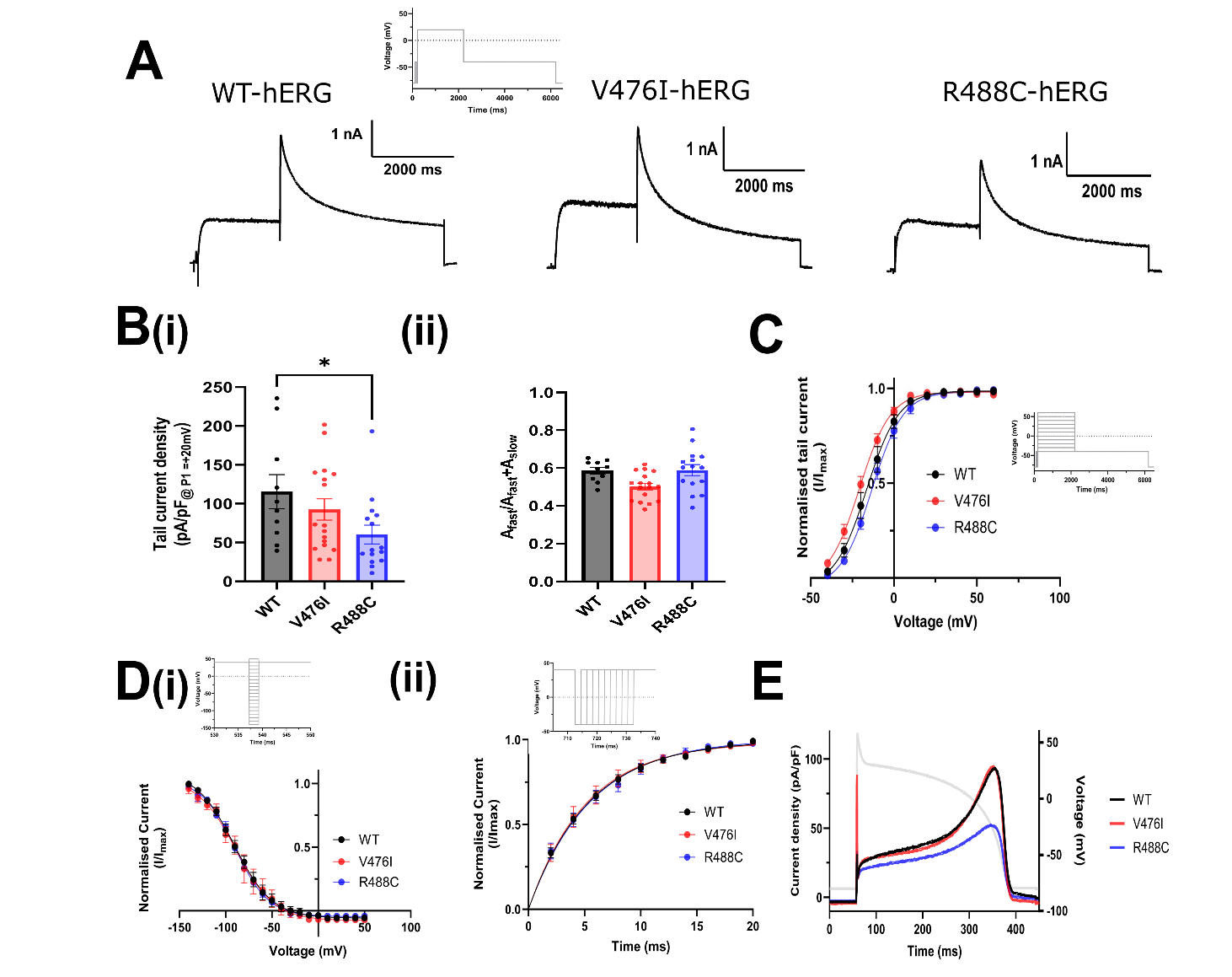
**

***Figure S2.* Electrophysiological characterisation of wild-type, V476I, and R488C hERG channels.** A, representative whole-cell hERG currents recorded from HEK-293 cells transiently overexpressing wild-type (WT), V476I, or R488C hERG channels. Currents were elicited with a depolarizing voltage step to +20 mV, followed by repolarization to –40 mV to evoke resurgent tail currents. B, (i) tail current density and (ii) proportion of deactivating tail current described by the deactivation τ_fast_, following biexponential fitting of the decaying tail current component. C, voltage dependence of activation, with tail currents normalized to the maximum current observed during the protocol, fitted with a Boltzmann function. D, (i) normalized voltage dependence of inactivation, fitted with a Boltzmann function, and (ii) recovery from inactivation, fitted with a one-phase decay function. E, representative hERG current traces recorded in response to a ventricular action potential waveform voltage command (overlaid in grey), applied at frequency of 1 Hz. p<0.05. Voltage protocols are shown as insets. Sample sizes (n) and quantified values of gating parameters are provided in Table S2.

*
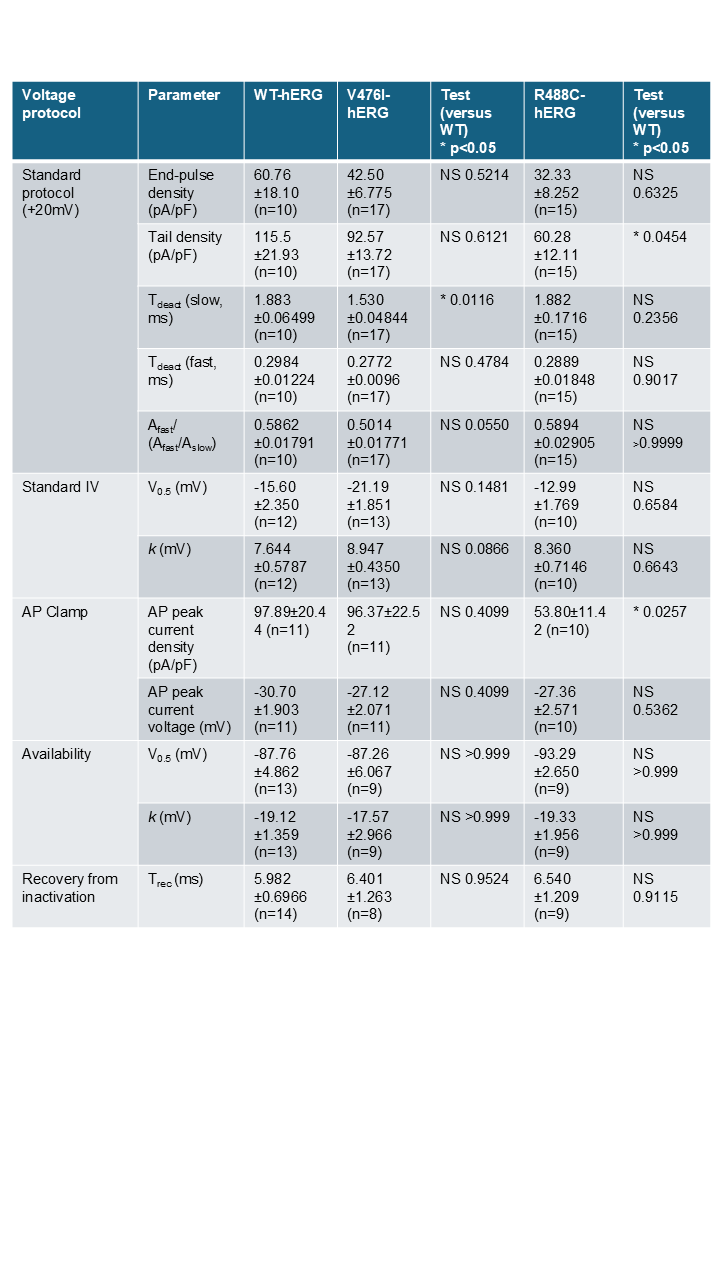
*

***Table S2:* Summary of WT, V476I and R488C-hERG electrophysiological characterisation.**

*Deactivation time constants (τ_slow_, τ_fast_) were acquired from fitting tail currents with a biexponential function. Activation/inactivation parameters (V_0.5_ and k) were obtained through fitting the normalised current-voltage relationship with standard Boltzmann functions. Recovery time constant (τ_rec_) was calculated from a one-phase decay fit. * indicates p<0.05. NS; Not significant (i.e. p>0.05). All data was normality tested to assess distribution. Where normality was passed, i.e. exhibited normal distribution, a one-way ANOVA was used with post-hoc Bonferroni correction. Where normality failed, a Kruskal–Wallis test was used instead, with post-hoc Bonferroni correction.*

**References**

1. O'Neill, M. J., Ng, C. A., Aizawa, T., Sala, L., Bains, S., Winbo, A. *et al.* (2024) Multiplexed Assays of Variant Effect and Automated Patch Clamping Improve KCNH2-LQTS Variant Classification and Cardiac Event Risk Stratification *Circulation* **150**, 1869-1881

2. Liang, Y., Yang, S., Zheng, L., Wang, H., Zhou, J., Huang, S. *et al.* (2022) Research progress of reduced amino acid alphabets in protein analysis and prediction *Comput Struct Biotechnol J* **20**, 3503-3510

3. Stephenson, J. D., and Freeland, S. J. (2013) Unearthing the root of amino acid similarity *J Mol Evol* **77**, 159-169

4. Zhang, Y., Grimwood, A. L., Hancox, J. C., Harmer, S. C., and Dempsey, C. E. (2022) Evolutionary coupling analysis guides identification of mistrafficking-sensitive variants in cardiac K(+) channels: Validation with hERG *Front Pharmacol* **13**, 1010119

5. Zhang, Y. H., Colenso, C. K., Sessions, R. B., Dempsey, C. E., and Hancox, J. C. (2011) The hERG K(+) channel S4 domain L532P mutation: characterization at 37 degrees C *Biochim Biophys Acta* **1808**, 2477-2487

6. Butler, A., Zhang, Y., Stuart, A. G., Dempsey, C. E., and Hancox, J. C. (2018) Action potential clamp characterization of the S631A hERG mutation associated with short QT syndrome *Physiol Rep* **6**, e13845

7. El Harchi, A., McPate, M. J., Zhang, Y. H., Zhang, H., and Hancox, J. C. (2010) Action potential clamp and mefloquine sensitivity of recombinant 'I KS' channels incorporating the V307L KCNQ1 mutation *J Physiol Pharmacol* **61**, 123-131

8. McPate, M. J., Zhang, H., Adeniran, I., Cordeiro, J. M., Witchel, H. J., and Hancox, J. C. (2009) Comparative effects of the short QT N588K mutation at 37 degrees C on hERG K+ channel current during ventricular, Purkinje fibre and atrial action potentials: an action potential clamp study *J Physiol Pharmacol* **60**, 23-41
